# Investigation of *Brassica juncea*, *Forsythia suspensa,* and *Inula britannica*: phytochemical properties, antiviral effects, and safety

**DOI:** 10.1186/s12906-019-2670-x

**Published:** 2019-09-11

**Authors:** Won-Young Bae, Hyeong-Yeop Kim, Kyoung-Sook Choi, Kyung Hoon Chang, Young-Ho Hong, Jongsu Eun, Na-Kyoung Lee, Hyun-Dong Paik

**Affiliations:** 1 Department of Food Science and Biotechnology of Animal Resources, Konkuk University, Seoul, South Korea; 2 CJ CheilJedang Blossom Park, Gyeonggi-do, South Korea

**Keywords:** Medicinal herbs, Phytochemical properties, Antiviral effect, Human safety

## Abstract

**Background:**

General antiviral agents such as oseltamivir are associated with certain adverse effects and the emergence of resistance. This study investigated the phytochemical properties, antiviral activities, and safety of three herbs used in traditional Korean medicine.

**Methods:**

Extracts of three medicinal herbs (*Brassica juncea*, *Forsythia suspensa,* and *Inula britannica*) were prepared using ethanol or water. The total phenolic, flavonoid, and saponin content, condensed tannin content, and reducing sugar content of the herb extracts were determined via phytochemical screening. Tandem mass analysis was performed using an ultra-performance liquid chromatography (UPLC)-electrospray ionization (ESI)-Q/Orbitrap instrument. Virus titrations were determined via tissue culture infective dose (TCID_50_) and cytotoxicity assays. Hemolysis and hepatotoxicity were measured to determine safety.

**Results:**

Among the three medicinal herbs, *F. suspensa* showed the highest concentration of phenolic compounds, flavonoids, and saponins. The number of phytochemical compounds detected via tandem mass analysis of *B. juncea, F. suspensa,* and *I. britannica* was 5 (including sinigrin, m/z [M-H] = 358.02), 14 (including forsythoside A, m/z [M-H] = 623.19), and 18 (including chlorogenic acid, m/z [M-H] = 353.20), respectively. The antiviral effects of the *B. juncea* extracts (ethanol and water) and *I. britannica* extract (ethanol) were further investigated. The ethanol extract of *B. juncea* showed a 3 Log TCID_50_/25 μL virus titration reduction and the water extract showed a selectivity index of 13.668 against infected influenza H1N1 virus A/NWS/33. The *B. juncea* extracts did not show hemolysis activities and hepatotoxicity (< 20%). The ethanol extract of *I. britannica* showed the most effective virus titration decrease, whereas its hemolytic and hepatotoxicity values were the most significantly different compared to the control. Despite the high concentration of phytochemicals detected in *F. suspensa*, the extract showed approximately 1 Log TCID_50_/25 μL at the highest concentration.

**Conclusion:**

*B. juncea* may show antiviral effects against H1N1 in a host. In addition, *B. juncea* may also show decreased disadvantages compared to other antiviral agents.

## Background

Medicinal herbs have been used for the treatment of various diseases in Korea, China, Japan, and other East Asian countries such as Malaysia and Vietnam [1]. In Korea, many traditional medicinal herbs have been researched and are used as edible medicines. For example, *Allium hookeri* root suppressed the lipopolysaccharide-induced expression of nuclear factor-kappa B (NF-κB) in RAW 264.7 cells [2], *Phragmitis rhizoma* reduced the myelotoxicity of docetaxel, a commonly used anticancer agent [3], *Rosa gallica* exhibited in vitro antioxidant and anti-skin aging effects as a matrix metalloproteinase-1 (MMP-1) inhibitor [4], and *Acer okamotoanum* prevented oxidative stress in SH-SY5Y neuronal cells [5].

The influenza virus thrives in a wide range of regions and hosts because of the occurrence of genetic recombination and cross species transmission of the influenza virus. This leads to huge economic losses in the poultry industry and threatens public health [6]. The influenza A virus subtypes H1N1 (A/H1N1) and H3N2 (A/H3N2) and influenza B virus have periodically spread in winter, causing more than 250,000 deaths [7]. Oseltamivir and zanamivir are antiviral agents approved by the United States (US) Food and Drug Administration (FDA) but these neuraminidase (NA) inhibitors cannot prevent the emergence of resistance [8]. M2 protein inhibitors such as amantadine and rimantadine are active only against the influenza A virus [9]. Therefore, novel antiviral agents are needed to counteract the disadvantages of existing antiviral agents.

*Brassica juncea* is a brown mustard seed that has a spicy flavor and is used as a condiment. In addition, it contains various bioactive chemicals and is inexpensive, and is therefore used in human foods and animal feeds [10]. *Forsythia suspensa* is known for its high saponin content and is used to treat various inflammatory symptoms, such as carbuncles or abscesses associated with swelling, common cold, and fever [11]. *Inula britannica,* a rich source of flavonoids [12–20], is used as a traditional medicine to treat bronchitis, digestive disorders, and inflammation in Korea [12]. Considering these phytochemical properties, its antimicrobial effects against *Helicobacter pylori* [21] and its potential as a food additive in cheddar-type cheese [22] have been studied previously. This study aimed to investigate the phytochemical properties and antiviral effects of these traditional Korean medicinal herbs. In addition, the hepatotoxicity and hemolytic activities of these plants extracts were evaluated to determine their safety.

## Methods

### Chemicals and medicinal herbs

Folin-Ciocalteu’s phenol reagent, vanillin, saponin from quillaja bark, and (+)-catechin were purchased from Sigma-Aldrich (St. Louis, MO, USA). Gallic acid was purchased from Tokyo Chemical Industry (Tokyo, Japan) and sodium carbonate was purchased from Samchun Chemical (Pyeongtaek, South Korea). Tamiflu were obtained from Roche (Seoul, Korea). *B. juncea* (seed), *F. suspensa,* (fruit) and *I. britannica* (seed) were obtained from Kyungdong-Market in Seoul, Korea. *B. juncea*, *F. suspensa,* and *I. britannica* were authenticated by Professor Hyun-Dong Paik at the Laboratory of Biotechnology (Konkuk University, Seoul, Korea) and stored as voucher specimen KU-H13, KU-H22 and KU-H26, respectively.

### Extraction

The medicinal herbs were extracted according to a method previously described, with some modifications [23]. The herb powder (100 g) was extracted with 1 L distilled water and ethanol (1:10 w/v) at 70 °C in a boiling pot (OCOO, Boryeong, South Korea) for 6 h. The extracts were filtered through Whatman No. 2 paper via vacuum filtration. After filtration, the extracts were stored at 4 °C. The soluble solid content of the extracts was measured as per the methods used by the Association of Official Analytical Chemists (AOAC) [24]. For quantification of phenolic compounds and cytotoxicity assay, all extracts were lyophilized before used.

### Phytochemical screening

The total phenolic, flavonoid, and saponin content, condensed tannin content, and reducing sugar content of the herb extracts were determined via phytochemical screening. The extracts were filtered through a 0.45-μm membrane filter and their phytochemical properties were evaluated.

Total phenolic content was determined via Folin-Ciocalteu assay with modifications [23]. The extracts (90 μL) were mixed with 1.8 mL of 2% (w/v) sodium carbonate solution and 90 μL of 50% (v/v) Folin-Ciocalteu’s reagent and incubated for 30 min. Molybdenum oxide content was measured via spectrophotometry (X-ma 3200, Human corporation, Seoul, Korea) at a wavelength of 752 nm. Gallic acid was used as the standard and the compounds’ phenolic content was expressed as gallic acid equivalents (mg GAE/g solid).

Total flavonoid content was measured via aluminium chloride assay [25]. The extracts (100 μL) were incubated with 20 μL of 5% sodium nitrite and 800 μLof 60% ethanol to determine flavonoid content. After 6 min, 20 μL of 10% aluminum chloride was added and 60 μL of 4% sodium hydroxide was added 6 min later. The mixtures were then incubated for 30 min. The absorbance of the flavonoid and aluminum chloride complex (yellow) was measured using a microplate reader (Molecular Devices, San Jose, CA, USA) at a wavelength of 405 nm. Quercetin was used as the standard and flavonoid content was expressed as quercetin equivalents (mg QE/g of solid).

Total saponin content was measured via the vanillin assay [26]. Briefly, 100 μL extracts were mixed with 100 μL of 8% (w/v) vanillin solution in methanol and 1 mL of 72% (v/v) sulfuric acid in methanol. The mixture was incubated at 60 °C for 10 min. After incubation, the mixture was cooled for 15 min, and the absorbance was measured using a microplate reader at a wavelength of 540 nm. Quillaja saponin was used as the standard and saponin content was expressed as quillaja saponin equivalents (mg QSE/g solid).

Condensed tannin content was measured via vanillin-HCl assay [25] with modifications. The reaction mixture comprised 20 μL extracts, 600 μL of 4% (w/v) vanillin solution in methanol, and 300 μL concentrated hydrochloric acid. The mixture was incubated at 25 °C in the dark. After 20 min, absorbance was measured at a wavelength of 500 nm using a spectrophotometer. (+)-Catechin was used as the standard and tannin content was expressed as catechin equivalents (mg CE/g solid).

Reducing sugar content was evaluated using 3,5-dinitrosalicylic acid (DNS) [27]. Briefly, 100 μL extracts were reacted with 100 μL DNS reagents for 10 min in boiling water. DNS solution was prepared by dissolving 2.5 g DNS in 25 mL distilled water at 80 °C. Potassium sodium tartrate (75 g) and 50 mL of 2 N sodium hydroxide solution was added to the cooled DNS solution. The final volume of DNS reagents was made up to a volume of 250 mL with distilled water. After the reaction, the mixtures were cooled on ice for 15 min and 1 mL distilled water was added. The absorbance was measured using a microplate reader at a wavelength of 540 nm. Glucose was used as the standard and reducing sugar content was expressed as glucose equivalents (mg GE/g solid).

### Ultra-performance (UPLC)-electrospray ionization (ESI)-Q/Orbitrap mass analysis

Tandem mass analysis was performed using a UPLC-ESI-Q/Orbitrap instrument [28]. The UPLC system (Ultimate 3000, Thermo Fisher Scientific, Waltham, MA, USA) was coupled to a Q-Exactive Orbitrap mass spectrometer (Thermo Fisher Scientific). The extracts were separated on a Hypersil GOLD™ C18 column (2.1 mm × 100 mm, 1.9 μm, Thermo Fisher Scientific) and ionized in negative mode.

The UPLC separation system comprised a binary solvent system (A, 0.1% formic acid in water, and B, 0.1% formic acid in acetonitrile) operating at a flow rate of 0.2 mL/min. The linear gradient used was as follows: 0–2.779 min (90–80% A, 10–20% B), 2.779–5.558 min (80% A, 20% B), 5.558–10.004 min (80–75% A, 20–25% B), 10.004–22.231 min (75–10% A, 25–90% B), 22.231–25.009 min (10–90% A, 90–10% B), and 25.009–31.000 min (90% A, 10% B). The injection volume was 1 μL. The following parameters were used: mass range, 100–1000 mass range; sheath gas flow rate, 40 arbitrary units (AU); auxiliary gas flow rate, 10 AU; heater temperature, 250 °C; capillary temperature, 320 °C; capillary voltage, − 3.5 V; and spray voltage, 2.5 kV. The resolution was set to 35,000 for full scan mass measurements and 17,500 for MS^2^ measurements. Data analysis was performed using Xcalibur™ software (Thermo Fisher Scientific).

### Cell culture and virus

Madin-Darby Canine Kidney (MDCK) cells were obtained from the American Type Culture Collection (ATCC, Manassas, VA, USA) and maintained in minimum essential medium (MEM, Hyclone™, Logan, UT, USA) supplemented with 10% (v/v) heat-inactivated fetal bovine serum (FBS, Hyclone™) and 1% (v/v) penicillin-streptomycin (Hyclone™) [8].

The human influenza H1N1 virus A/NWS/33 was propagated in allantoic fluid (AF) obtained from 9- to 11-day-old embryonated chicken eggs for 48 h at 37 °C. After inoculation, virus-infected AF was harvested and stored at − 80 °C until further use [29].

### Tissue culture infective dose (TCID_50_) determination

The TCID_50_ was evaluated in MDCK cells seeded in 96-well plates [8]. Equal volumes of medicinal extracts and viruses were mixed and incubated at 4 °C for 30 min. After incubation, the mixture was added to MDCK cells seeded at a density of 2 × 10^4^ cells/well. The cells were incubated for 4 to 5 days at 37 °C and the cytopathic effect (CPE) was evaluated using 1% crystal violet solution.

### Cytotoxicity assay

The 50% cytotoxic dose (CC_50_) and the 50% effective concentration (EC_50_) were measured to calculate the selectivity index (SI) [30]. MDCK cells were pre-incubated in 6-well plates until the formation of a monolayer. MDCK cell lines were infected influenza by incubating for 40 min. After infection, remaining viruses were removed and infected cells were incubated with 3 mL medium containing 1% agarose and extracts for 48 h at 37 °C in 5% CO_2_. After incubation, the cells were stained with 1% crystal violet solution to evaluate the presence of plaques. Cell viability was measured using neutral red dye (0.034%) and cells were stained for 2 h at 37 °C before extracting dye using ethanol-Sorenson citrate buffer (1:1) for 30 min in the dark. Absorbance was measured using a microplate reader at a wavelength of 540 nm. The SI was calculated by dividing the CC_50_ by the EC_50_.

### Hepatotoxicity and hemolysis

To determine the safety of the antiviral agents studied, the hepatotoxicity and hemolysis of each extract were evaluated. HepG2 cells (hepatocellular carcinoma cells) were obtained from the Korean Cell Line Bank (KCLB, Seoul, Korea). The cells were maintained in MEM containing 10% (v/v) FBS and 1% (v/v) penicillin-streptomycin solution in a humidified atmosphere containing 5% CO_2_ at 37 °C. Defibrinated sheep blood was obtained from Kisanbio (Seoul, Korea).

Hepatotoxicity was evaluated via MTT assay [31]. HepG2 cells were seeded at a density of 10^5^ cells/well in a 96-well microplate. After 20 h, the cells were treated with extracts and incubated for 48 h. After incubation, the medium was replaced with 2.5 mg/mL MTT solution and incubated to allow the reduction of tetrazolium to formazan. After 2 h, formazan was dissolved in 100 μL dimethyl sulfoxide (DMSO) and the absorbance was measured using a spectrophotometer at a wavelength of 570 nm. Cell viability was calculated according to the following formula:

$$ \mathrm{Cell}\ \mathrm{viability}\ \left(\%\right)=\left(\frac{{\mathrm{A}}_{\mathrm{sample}}}{{\mathrm{A}}_{\mathrm{control}}}\right)\times 100 $$

where A_control_ is the absorbance of the control (without extract) and A_sample_ is the absorbance of extract-treated samples.

The extracts’ hemolytic activities were evaluated in sheep blood [32]. Briefly, 100 μL extracts were added to 875 μL phosphate-buffered saline (PBS). Sheep blood (25 μL) was then added and incubated at 37 °C for 30 min. After incubation, all mixtures were centrifuged at 5500×*g* for 1 min at 4 °C. Hemolytic activity was assessed by measuring the optical density of the supernatant at a wavelength of 540 nm. Hemolytic activity was calculated using to the following formula:

$$ \mathrm{Hemolytic}\ \mathrm{activity}\ \left(\%\right)=\left(\frac{{\mathrm{A}}_{\mathrm{sample}}}{{\mathrm{A}}_{\mathrm{control}}}\right)\times 100 $$

where A_control_ and A_sample_ are the absorbance of the positive control and extracts, respectively. The lysis buffer (positive control) comprised 0.1 mM EDTA and 0.5% Triton X-100 in 50 mM potassium phosphate buffer (pH 7.4). PBS was used as the negative control.

### Statistical analysis

Statistical analysis was performed using the IBM SPSS Statistics version 18 software (IBM, New York, NY, USA). Two independent samples (containing controls) were compared by *t*-test at significant level (*p* < 0.05).

## Results

### Phytochemical screening and tandem mass analysis

The phytochemical properties and soluble solid concentrations of the three medicinal herbs are indicated in Table 1. The ethanol and water extracts of *B. juncea* comprised 62.6 mg QE/g solid and 62.7 mg QSE/g solid, respectively. *F. suspensa* was a rich source of phytochemicals and contained the following: phenols, 147.4 mg GAE/g solid; flavonoids, 242.3 mg QE/g solid; and saponins, 439.3 mg QSE/g solid. The ethanol extract of *I. britannica* contained 225.7 GAE/g solid (phenols) and 288.1 mg QSE/g solid (flavonoids). The following were detected in the *I. britannica* water extract: phenols, 50.8 GAE/g solid; flavonoids, 51.6 mg QE/g solid; and saponins, 82.9 mg QSE/g solid. All extracts showed low levels of condensed tannins and reducing sugars. Among the three medicinal herbs, *F. suspensa* showed the highest soluble solid content.

**Table 1:** Phytochemical screening of *Brassica juncea*, *Forsythia suspensa,* and *Inula britannica* extracts

Plants	Extract Solvents	Total phenolic contents (mg GAE/g solid)	Total flavonoid contents (mg QE/g solid)	Total saponin contents (mg QSE/g solid)	Condensed tannin contents (mg CE/g solid)	Reducing sugar contents (mg GE/g solid)	Soluble Solid Contents (mg/mL)
*Brassica juncea*	Ethanol	1.4 ± 0.1	62.6 ± 3.4	23.5 ± 1.4	1.4 ± 0.1	0.7 ± 0.0	14.1 ± 0.4
	Water	17.9 ± 0.1	4.1 ± 0.0	62.7 ± 0.3	0.4 ± 0.0	3.5 ± 0.2	0.3 ± 0.2
*Forsythia suspensa*	Ethanol	147.4 ± 5.8	242.3 ± 3.5	439.3 ± 4.9	14.1 ± 0.7	8.7 ± 0.9	25.8 ± 0.3
	Water	113.3 ± 4.3	40.7 ± 0.2	137.1 ± 2.2	0.7 ± 0.0	7.6 ± 0.2	42.4 ± 0.3
*Inula britannica*	Ethanol	42.1 ± 3.3	225.7 ± 5.1	288.1 ± 7.8	10.2 ± 0.3	4.5 ± 1.6	18.0 ± 0.7
	Water	50.8 ± 2.5	51.6 ± 1.0	82.9 ± 2.6	0.8 ± 0.0	5.0 ± 0.2	12.8 ± 0.3

Data are shown as means ± standard deviations of three independent experiments

The tandem mass analysis of the medicinal herb extracts is shown in Table 2. The phenolic and other phytochemical compounds detected here are in accordance with the results reported in previous studies [12–20, 33–42]. Five compounds were detected in the *B. juncea* extracts*,* including sinigrin, a member of the glucosinolate family [34]. Fourteen compounds were detected in the *F. suspensa* extracts, including caffeic acid (a member of the hydroxycinnamic acids class), quercetin, and kaempferol (flavonols). 6-Methoxyluteolin was not previously reported in *F. suspensa* but its presence was assumed from the m/z [M-H] and MS^2^ fragments of *I. britannica*. In *I. britannica*, 18 compounds were identified, including chlorogenic acid (hydroxycinnamic acids) and patuletin (flavonols). Rutin and hispidulin were also detected in the *I. britannica* extract.

**Table 2:** Ultra performance liquid chromatography (UPLC)-electrospray ionization (ESI)-Q/Orbitrap tandem mass analysis of medicinal herbs

Medicinal herbs	Extract Solvents	Compounds	Retention time (min)	m/z [M-H]	MS^2^ fragment	Molecular formula (Neutral form)	Contents (μg/mg)	References
*Brassica juncea*	Ethanol	Sinigrin	1.14	358.02533	96.95830	C_10_H_17_NO_9_S_2_	1.458 ± 0.027	[33, 34]
		Chlorogenic acid	18.03	353.20108	96.95840	C_16_H_18_O_9_	< LOQ^a^	[35]
	Water	Sinigrin	1.18	358.02857	96.95876	C_10_H_17_NO_9_S_2_	4.116 ± 0.129	[33, 34]
		*р*-Coumaric acid	5.67	163.03907	119.04838	C_9_H_8_O_3_	< LOQ	[35]
		Kaempferol	13.43	285.04058		C_15_H_10_O_6_	< LOQ	[35, 36]
		Chlorogenic acid	21.86	353.20112	96.95842	C_16_H_18_O_9_	< LOQ	[35]
*Forsythia suspensa*	Ethanol	Caffeic acid	1.05	179.03373	135.04332	C_9_H_8_O_4_	< LOQ	[37]
		Arctigenin	1.08	371.12045	325.18523	C_21_H_24_O_6_	2.270 ± 0.122	[38, 39]
		Vanillic acid	1.21	167.03438	123.04418, 108.02051	C_8_H_8_O_4_	0.653 ± 0.131	[37, 39]
		Protocatechuic acid	2.18	153.01786	109.02791	C_7_H_6_O_4_	0.426 ± 0.026	[39, 40]
		Astragalin	3.21	447.09172	269.10268	C_21_H_20_O_11_	0.730 ± 0.027	[38]
		Forsythoside A	3.48	623.19776	179.03370	C_29_H_36_O_15_	168.735 ± 1.549	[38–40]
		Chlorogenic acid	3.71	353.23580	191.05486, 96.95844	C_16_H_18_O_9_	0.351 ± 0.010	[39, 41]
		Rutin	4.56	609.14181	300.02756	C_27_H_30_O_16_	< LOQ	[39–41]
		Forsythoside G	6.26	769.25908	179.03370	C_35_H_46_O_19_	< LOQ	[39]
		Quercetin	12.27	301.03568	178.99739, 151.00303	C_15_H_10_O_7_	0.245 ± 0.054	[37–40]
		Kaempferol	12.56	285.04062		C_15_H_10_O_6_	< LOQ	[38–40]
	Water	Forsythoside A	6.10	623.19745	179.03355	C_29_H_36_O_15_	78.735 ± 1.703	[38–40]
		Chlorogenic acid	27.76	353.20124	96.95845	C_16_H_18_O_9_	0.530 ± 0.014	[39, 41]
*Inula britannica*	Ethanol	Protocatechuic acid	0.03	153.01790	109.02793	C_7_H_6_O_4_	0.428 ± 0.010	[13]
		Caffeic acid	0.99	179.03378	135.04408	C_9_H_8_O_4_	< LOQ	[13]
		Chlorogenic acid	1.15	353.20122	191.05492, 96.95845	C_16_H_18_O_9_	0.579 ± 0.019	[14]
		Ergolide	1.17	305.07113	96.95837	C_17_H_22_O_5_	< LOQ	[13, 15, 42]
		Syringic acid	1.47	197.80735	151.06027	C_9_H_10_O_5_	< LOQ	[13]
		Isoquercetin	2.81	463.08749	300.02766	C_21_H_20_O_12_	< LOQ	[16]
		Nepitrin	4.80	477.10246	315.05086	C_22_H_22_O_12_	< LOQ	[13, 17]
		Rutin	5.83	609.14910	300.02769	C_27_H_30_O_16_	0.664 ± 0.010	
		Quercetin	5.84	301.03572	178.99742, 151.00220	C_15_H_10_O_7_	6.142 ± 0.390	[13, 15, 18, 19]
		Kaempferol	6.34	285.04067		C_15_H_10_O_6_	0.221 ± 0.025	[12, 13, 17, 18]
		6-Methoxyluteolin	6.50	315.05071	300.02767	C_16_H_12_O_7_	4.261 ± 0.137	[15, 17, 19]
		Patuletin	9.60	331.04501	316.02324, 285.04065	C_16_H_12_O_8_	< LOQ	[12, 13, 20]
		Ferulic acid	10.92	193.04926	147.02829	C_10_H_10_O_4_	0.865 ± 0.012	[13]
		Hispidulin	13.60	299.05650	284.03236	C_16_H_12_O_6_	0.867 ± 0.083	
	Water	Chlorogenic acid	0.02	353.20114	191.05487	C_16_H_18_O_9_	< LOQ	[14]
		Caffeic acid	0.11	179.03377	135.04334	C_9_H_8_O_4_	2.396 ± 0.028	[13]
		Ergolide	0.95	305.06863	96.95835	C_17_H_22_O_5_	< LOQ	[13, 15, 42]
		Quercetin	1.02	301.20163	178.99738, 151.00216	C_15_H_10_O_7_	< LOQ	[13, 15, 18, 19]
		Vanillic acid	1.41	167.03338	123.04357	C_8_H_8_O_4_	0.824 ± 0.028	[13]
		Protocatechuic acid	2.03	153.01789	109.02792	C_7_H_6_O_4_	0.177 ± 0.025	[13]
		6-Methoxyluteolin	2.50	315.05063	300.02761	C_16_H_12_O_7_	< LOQ	[15, 17, 19]
		Kaempferol	13.36	285.04061		C_15_H_10_O_6_	< LOQ	[12, 13, 17, 18]

Experiments were conducted in triplicate

Quantification of phytochemical compounds are shown as means ± standard deviations of three independent experiments

^a^LOQ, limit of quantification

### Antiviral effects

The virus titration results of the medicinal herb extracts are shown in Table 3. The ethanol extract of *B. juncea* showed approximately 3 Log TCID_50_/25 μL reduction at the highest concentration. Whereas the water extract of *B. juncea* did not show reduction of virus titer comparing to control. In the ethanol extract of *I. britannica*, no virus was detected at the highest concentration and a titer of 2.5 Log TCID_50_/25 μL was observed after a 10-fold dilution of the extract. Water extract of *I. britannica* reduced approximately 1 Log TCID_50_/25 μL of virus titer at highest concentration but low concentration of water extract of *I. britannica* were not effective in H1N1 virus A/NWS/33. All *F. suspensa* extracts caused a 12.59% decrease in the virus titers (3.9 Log TCID_50_/25 μL) although these were considered ineffective compared to the *B. juncea* and *I. britannica* extracts.

**Table 3:** Virus titration of medicinal herb extracts against influenza H1N1 virus A/NWS/33

Plants	Extract Solvents	Virus titration (Log TCID_50_^a^/25 μL)				
		Total dilution (−fold)				
		Control	100	50	10	1^b^
*Brassica juncea*	Ethanol	4.8	4.6	4.1^**^	3.6^**^	1.5^**^
	Water	4.8	4.9	4.8	4.9	4.6
*Forsythia suspensa*	Ethanol	4.8	4.6	3.9^**^	4.0^**^	3.5^**^
	Water	4.8	4.6	4.3^**^	3.9^**^	3.8^**^
*Inula britannica*	Ethanol	4.8	4.5	4.1^**^	2.5^**^	0^**^
	Water	4.8	4.9	4.9	4.6	3.9^**^

^a^TCID_50_, median tissue culture infective dose

^b^Soluble solid concentration (*B. juncea* ethanol extract, 14.1 mg/mL.; *B. juncea* water extract, 0.3 mg/mL; *F. suspensa* ethanol extract, 25.8 mg/mL; *F. suspensa* water extract 42.4 mg/mL; *I. britannica* ethanol extract 18.0 mg/mL; *I. britannica* ethanol extract 12.8 mg/mL)

Experiments were conducted in triplicate

Significant differences compared to control are indicated by asterisks (**; *p* < 0.01)

The antiviral effects of the extracts against virus-infected cells are reported in Table 4. The water extract of *B. juncea* showed a CC_50_ of 9.73 mg and an EC_50_ of 0.71 mg (SI = 13.668). The other extracts did not show significant antiviral effects on virus-infected cells. In addition, all extract of *F. suspensa* and *I. britannica* were showed higher toxicity than extract of *B. juncea*.

**Table 4:** Cytotoxicity, antiviral effect, and selectivity index of medicinal herb extracts

Materials		CC_50_^a^ (μg/mL)	EC_50_^b^ (μg/mL)	SI^c^
Tamiflu		569.25 ± 13.43	1.72 ± 0.21	330.170
Chlorogenic acid		72.34 ± 2.21	24.77 ± 1.03	2.920
Kaempferol		18.63 ± 0.06	2.46 ± 0.21	7.585
Plants	Extract Solvents	CC_50_ (mg/mL)	EC_50_ (mg/mL)	SI
*Brassica juncea*	Ethanol	1.91 ± 0.11	Not effective	Not effective
	Water	9.73 ± 1.40	0.71 ± 0.06	13.668
*Forsythia suspensa*	Ethanol	0.02 ± 0.00	Not effective	Not effective
	Water	0.10 ± 0.02	Not effective	Not effective
*Inula britannica*	Ethanol	0.19 ± 0.02	Not effective	Not effective
	Water	0.68 ± 0.11	Not effective	Not effective

^a^CC_50_, 50% cell cytotoxicity concentration

^b^EC_50_, 50% virus-inhibitory concentration

^c^SI, selectivity index (CC_50_/EC_50_)

Experiments were conducted in triplicate

### Safety test

**Fig. 1:**
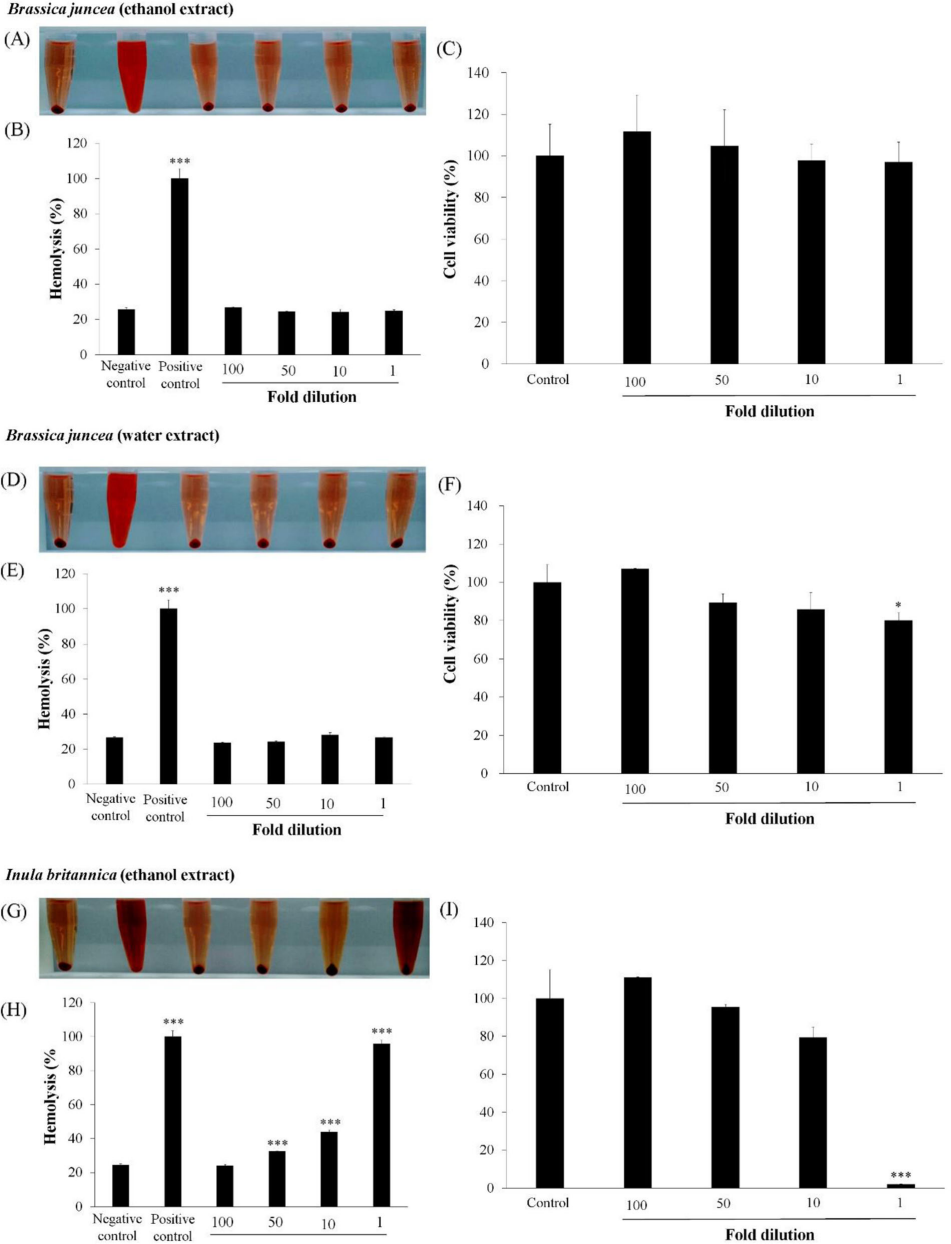
Hemolytic and cytotoxicity of medicinal herb extracts. (**a**, **d**, and **g**) Qualitative analysis of hemolysis; (**b**, **e**, and **h**) Quantitative analysis of hemolysis; (**c**, **f**, and **i**) Viability of HepG2 cells. The soluble solid concentrations of extracts (×1) were 14.1 (*Brassica juncea* ethanol extract), 0.3 (*Brassica juncea* water extract), and 18.0 mg/mL (*Inula britannica* ethanol extract). Data are shown as means ± standard deviations of three independent experiments. ^*^p < 0.05 and ^***^p < 0.001 indicated significant differences compared to the negative control

The hemolytic activities and hepatotoxicity of the medicinal herb extracts are shown in Fig. 1. The *B. juncea* extracts (ethanol and water) did not show significant hemolytic activity at any dilution. The *I. britannica* extract showed 24.03, 32.48, 43.86, and 95.85% hemolysis at 100-, 50-, 10-, and 1-fold dilutions, respectively. The hemolytic activities of the extracts used at 50-, 10-, and 1-fold dilutions were significantly greater than that of the negative control (24.40%, *p* < 0.001).

The viability of cells treated with 100-, 50-, 10-, and 1-fold dilutions of *B. juncea* ethanol extract was 111.71, 104.71, 97.71, respectively, and 96.89%, and viability was 107.14, 89.42, 85.89, and 79.97% in cells treated with the water extract, respectively. *I. britannica* extract treatment (100-, 50-, and 10-fold dilutions) resulted in 110.92, 95.34, and 75.36% cell viability, respectively. High toxicity (2.09% viability) was observed in cells treated with the extract diluted 1-fold (*p* < 0.05).

## Discussion

Phenolic compounds are commonly found in fruits, vegetables, grains, herbs, and spices. Phenolic acids, stilbenes, flavonoids, lignans, and ellagic acids are phenolic compounds found in plant foods. The bio-functionalities of these compounds have been studied and they can be used to treat various diseases and disorders without adverse effects [43]. Various phenolic compounds also show antiviral effects [8, 30, 44–47]. Chlorogenic acid, a caffeoylquinic acid, showed inhibitory effects on NA and H1N1 infection [45]. Quercetin [46, 47], kaempferol [46, 47], isorhamnetin [46], rutin [47], and isoquercetin [47] showed antiviral effects by suppressing viral mRNA expression, hemagglutinin (HA), and NA. Moreover, kaempferol attenuated inflammatory symptoms and decreased mortality in H9N2-infected mice [48]. In this study, the phytochemical properties and antiviral effects of three medicinal herbs were investigated. Five compounds were identified from *B. juncea*, including chlorogenic acid and kaempferol, and 18 compounds were detected in *I. britannica*, which included the antiviral phenolic compounds mentioned above (Table 2).

Several studies have reported the use of medicinal herbs in various forms including solvent extracts [44, 49, 50], essential oils [51], and powders [52]. Ghoke et al. [49] reported that hydro-methanol leaf plant extracts decreased HA titers and virus genome copy numbers. Hossan et al. [44] confirmed that embelin, the most abundant compound in *Embelia ribes* extract, was able to dock with HA, thus hindering the binding of HA to sialic acid-glycoprotein receptors on the host cells. In addition, Tang et al. [52] demonstrated that a mixture of medicinal herb powders inhibited influenza A virus H5N1 infection in mice. The benefits of medicinal herbs are now widely recognized and the demand for natural medicines has increased [53], requiring further research into medicinal herbs containing antiviral agents.

Oseltamivir, which is generally used to treat influenza, causes adverse effects such as nausea and vomiting [54, 55]. In severe cases, enterorrhagia, alimentary tract hemorrhage, and liver injury occurred after treatment with oseltamivir. Feng et al. [54] reported that a 6-year-old boy treated with 60 mg oseltamivir twice a day showed increased alanine transaminase (ALT) and aspartate transaminase (AST) levels. In addition, bilirubin content was increased by liver damage. Powder formulation of zanamivir, another NA inhibitor, was reportedly well tolerated, although inhalation resulted in low bioavailability [56, 57]. Conversely, intravenous administration of aqueous zanamivir resulted in higher bioavailability but this was accompanied by severe adverse effects [56]. Furthermore, Kiatboonsri et al. [58] reported nebulization treatment with zanamivir caused fatal respiratory events in a 25-year-old pregnant woman. In the current study, the hepatotoxicity and hemolytic activities of three medicinal herb extracts showing antiviral effects were measured to assess safety. The ethanol extract of *B. juncea* did not show hepatotoxicity or hemolytic activity, but decreased virus titers from 4.6 to 1.5 Log TCID_50_/25 μL. Treatment with the water extract of *B. juncea* resulted in 80% cell viability, and no hemolytic activity was observed at the highest treatment concentration. Furthermore, the SI was 13.668 when cells were treated with the CC_50_ (9.73 mg). Ding et al. [45] reported that the SI of chlorogenic acid was 8.12 and Dayem et al. [46] reported that the SI of kaempferol in H1N1-infected MDCK cells was 7. In this study, chlorogenic acid and kaempferol of SI were measured 2.920 and 7.585, respectively. By comparing the SI of *B. juncea* extract with those of chlorogenic acid and kaempferol, we can conclude that the extract showed higher antiviral effects, as it contains phenolic compounds as well as both chlorogenic acid and kaempferol. This suggests the potential of *B. juncea* as a potent antiviral agent.

## Conclusions

The phytochemical properties and antiviral effects of three medicinal herbs were analyzed. Two antiviral compounds (chlorogenic acid and kaempferol) were detected in *B. juncea*, and six antiviral phenolic compounds were identified in *I. britannica*. The SI of the water extract of *B. juncea* was higher than those of chlorogenic acid and kaempferol. Moreover, *B. juncea* did not show hemolytic activity and hepatotoxicity. These properties suggest the potential of *B. juncea* as an antiviral agent.

## Data Availability

The datasets used and/or analyzed during the current study are available from the corresponding author on reasonable request.

## Abbreviations

AF: Allantoic fluid
ALT: Alanine transaminase
AST: Aspartate transaminase
ATCC: American Type Culture Collection
CC: Cytotoxic concentration
CPE: Cytopathic effect
DMSO: Dimethyl sulfoxide
DNS: (3,5-dinitrosalicylic acid)
EC: Effective concentration
FBS: Fetal bovine serum
FDA: United States (US) Food and Drug Administration
HA: Hemagglutinin
KCLB: Korean Cell Line Bank
MDCK: Madin-Darby Canine Kidney
MEM: Minimum essential medium
MTT: 3-(4,5-dimethylthiazol-2-yl)-2,5-diphenyltetrazolium bromide
NA: Neuraminidase
PBS: Phosphate-buffered saline
SI: Selectivity index
TCID_50_: Tissue culture infective dose at 50%
